# Blood pressure, left ventricular structure and cognitive development in young children: a cross-sectional study

**DOI:** 10.1007/s12519-026-01078-7

**Published:** 2026-08-14

**Authors:** Hua-Lin Wang, Bo-Wen Du, Yue Zhou, Zhuo-Yan Li, Qian-Chuo Wang, Cai-Fang Xu, Zhi-Kang Xu, Xi Zhang, Sun Chen, Qian Chen, Kun Sun, Jian Wang

**Affiliations:** 1 Department of Pediatric Cardiology, Xinhua Hospital Affiliated to Shanghai Jiao Tong University School of Medicine, No. 1665, Kongjiang Road, Yangpu District, Shanghai, 200092 China; 2 Institute of Cardiovascular Development and Regeneration, Xinhua Hospital Affiliated to Shanghai Jiao Tong University School of Medicine, Shanghai, China; 3 Clinical Research Unit, Xinhua Hospital Affiliated to Shanghai Jiao Tong University School of Medicine, Shanghai, China; 4 Ministry of Education-Shanghai Key Laboratory of Children’s Environmental Health, Xinhua Hospital Affiliated to Shanghai Jiao Tong University School of Medicine, No. 1665, Kongjiang Road, Yangpu District, Shanghai 200092, China

**Keywords:** Blood pressure, Cognitive development, Left ventricular structure

## Abstract

**Background:**

As a consequence of hypertension, left ventricular (LV) structural alterations are associated with cognitive decline in the elderly. However, its role in early childhood remains unclear. We examined the relationship between blood pressure (BP), LV structure, and cognitive function in preschool-aged children. This population is free from aging-related comorbidities.

**Methods:**

We recruited 1279 4-year-old children from the Shanghai Birth Cohort (2018–2021), excluding those with birth defects or developmental delays. BP and echocardiographic assessment were conducted at follow-up. Cognitive function was evaluated using the Wechsler preschool and primary scales of intelligence-fourth edition.

**Results:**

Elevated BP (107.4 ± 6.2 mmHg) compared to normal BP (96.0 ± 5.9 mmHg) tended towards lower full scale intelligence quotient (IQ) [adjusted mean difference (*β*) = − 2.95, 95% confidence interval (CI) = − 4.65, − 1.25], processing speed index (*β* = − 1.77, 95% CI = − 3.41, − 0.14), working memory index (*β* = − 2.79, 95% CI = − 4.70, − 0.88) and visual spatial index (*β* = − 2.64, 95% CI = − 4.74, − 0.55). These associations persisted after adjusting for LV mass index (LVMI). Higher systolic BP, analyzed as a continuous variable, was associated with lower full scale IQ (*β* = − 0.12, 95% CI = − 0.21, − 0.03) and visual spatial index (*β* = − 0.23, 95% CI = − 0.35, − 0.10); diastolic BP showed the same trend. There was no significant association between cognitive function and LV structure or the mediating effect of LVMI.

**Conclusions:**

Higher BP was associated with poorer cognitive development as early as age four; LV structural alterations were not. This suggests that the relationship between BP and cognitive function was independent of LVMI in early childhood.

**Graphical abstract:**

**Figure:**
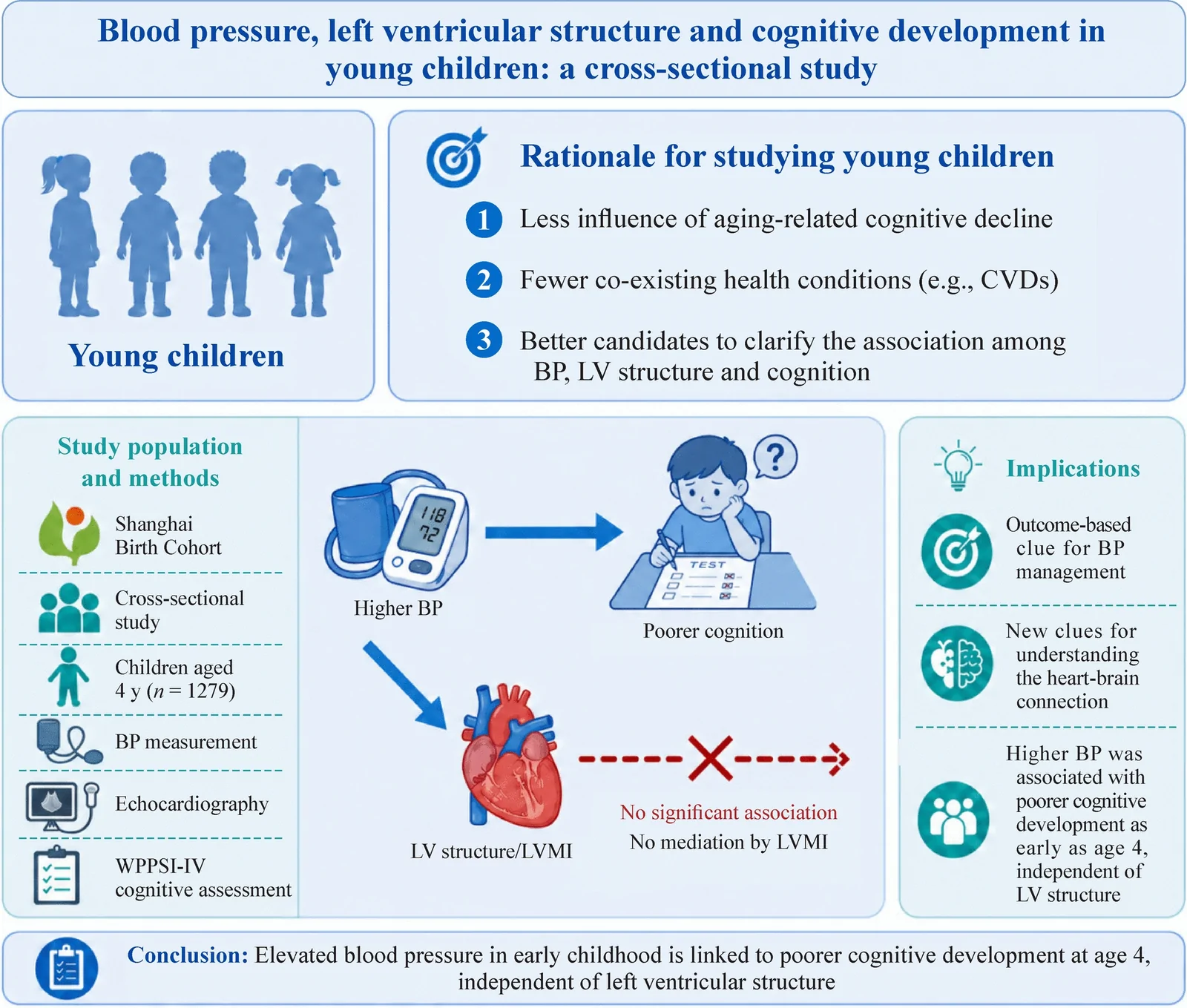

**Supplementary Information:**

The online version contains supplementary material available at https://doi.org/10.1007/s12519-026-01078-7.

## Introduction

High blood pressure (BP) in midlife or older age is a well-known risk factor for cognition decline and dementia [1]. As hypertensive end-organ damage may occur at a young age, decreased cognitive function has also been found in school-aged children with elevated BP [2, 3]. However, few studies have explored these associations during early childhood which represents a critical window for brain development. During this period cerebral blood flow regulation and neurovascular coupling are still maturing [4]. These processes may be particularly vulnerable to subtle elevations in BP, with implications for cognition development.

As a pathological increase in left ventricular mass (LVM), left ventricular hypertrophy (LVH) has been proposed as a common hypertensive target [5]. The increase in LVM was found to be associated with elevated risk of cognitive impairment [6–8] and cerebral small vessels disease [9] in the elderly; this was independent of BP. However, several studies found that cardiac remodeling or left ventricular geometric adaptation partially mediated the association between cumulative exposure to elevated BP and cognitive function [10, 11]. To date, the relationships between BP, LV structure, and cognitive function remain controversial.

Understanding this association might facilitate therapeutic target identification and reduce the risk of cognitive impairment. To address the question of whether lowering BP could reduce the risk of cognitive impairment or slow cognitive decline, several randomized controlled trials of BP management were conducted with inconclusive results [12–15]. In addition, most studies exploring mechanisms linking BP and cognitive function have focused on cerebrovascular disease and dementia pathology, leaving the role of cardiac structural changes largely unexplored [16].

The associations described above were explored in the elderly. This population usually has cardiovascular co-morbidities and underlying cerebral pathology, along with age related confounders that are difficult to control. Furthermore, processes that underly LV structural changes generally evolve over years. Hence, the relevance of these mechanisms to preschool-aged children is questionable. It follows that studies in young children would be a better model for study these.

To address these uncertainties we conducted the first study to examine the relationship between blood pressure, LV structure and cognitive function in a multi-centered preschool age population. This study might provide perspectives on heart–brain connections.

## Methods

Ethical approval for this study was granted by the Ethics Committee of Xinhua Hospital affiliated to Shanghai Jiao Tong University School of Medicine (No. XHEC-C-2013-001-2). Parents or legal guardians of all enrolled children provided written informed consent.

### Study design

We used the Shanghai Birth Cohort (SBC) in this study. Details of this cohort have been published previously [17]. There were 4127 couples initially enrolled in the cohort and there were 3692 live births. At enrollment, social-demographic information including parental age, ethnicity, educational level, occupation and maternal annual income was collected through a set of standardized questionnaires. Information regarding pregnancy and birth was obtained from medical records. Cardiovascular evaluation and assessment of cognitive development was performed from 2018 to 2021 for children at 4-years of age. Children with birth defects or delayed mental development were excluded. A total of 1279 children with cardiovascular data and complete intelligence quotient (IQ) assessment were finally included in the current study.

### Blood pressure and echocardiography measurement

BP was measured using an automatic BP monitor (OMRON HBP-1300; Omron Healthcare, Guangzhou, China), according to 2017 guidelines of the American Academy of Pediatrics [18]. Three readings were taken from the left arm in a resting supine position over 5 minutes; the mean of the lower two were used. The diagnostic cutoff for elevated blood pressure was defined as the 90th percentile of age-, sex-, and height-specific blood pressure reference values for Chinese children as updated in 2017 [19].

Echocardiography was performed using the EPIQ 7C ultrasound system with S8-3 (8–3 MHz) and X5-1 (1–5 MHz) transducers (Philips Healthcare, Andover, USA). Detailed methodology is described in our previous studies [20, 21]. Briefly, M-mode, 2-dimensional, and Doppler echocardiographic images were obtained from multiple views. Linear measurement of the LV including internal diameter, interventricular septal thickness (IVS), and posterior wall thicknesses (LVPW) were measured at end-diastole and end-systole according to American Society of Echocardiography guideline [22]. The LVM was determined using the anatomically validated formula of Devereux [23] and the LVM index (LVMI) was calculated by dividing LVM by height^2.7^ [24]. Relative wall thickness (RWT) was calculated using the following formula: (LVPW at end-diastole + IVS at end-diastole)/LV diastolic diameter [25]. Sex-specific 95th percentiles of LVMI derived from the cohort was used as the cutoff for LVH as there was no recommended standard on LVMI for 4-year-old children in China [26].

### Assessment of cognitive development

Cognitive development was quantified using the Wechsler primary and preschool scales of intelligence-fourth edition (WPPSI-IV) [27]. This is widely used to measure intellectual abilities in children from 2 years and 6 months to 7 years and 3 months. The WPPSI-IV has been translated into Chinese by the Beijing Normal University and revised over 10 years to ensure that the vocabulary was appropriate for Chinese children [28]. Five different subscales were evaluated: verbal comprehension index (VCI), visual spatial index (VSI), fluid reasoning index (FRI), working memory index (WMI), and processing speed index (PSI). Full-scale IQ (FSIQ) was calculated as a weighted composite of the five index scores (VCI, VSI, FRI, WMI, and PSI) according to the WPPSI-IV manual. Tests were performed by trained neurodevelopmental pediatricians who were unaware of participant BP and LV structure. Cognitive test scores were derived based on age-standardized Chinese norms of the WPPSI-IV, with high scores indicating better performance.

### Statistical analysis

Continuous variables were reported as means with standard deviation (SD) and categorical variables were reported as numbers with percentages. Comparisons of IQ were performed in different groups using a Student’s *t* test or one-way analysis of variance (one-way ANOVA). Bonferroni post hoc test was used for multiple pairwise comparisons. Multiple linear regressions were used to analyze associations between BP, LV structure and IQ. To explore the independent role of BP or LV structure in the association with IQ, linear regression models were used in different BP or LV structure subgroups. An interaction term was included in multiple linear regressions to examine any potential interaction between BP and LVH. Mediation analysis was performed to examine whether LVMI mediated the association between BP levels and WPPSI-IV scores. To flexibly model associations between BP or LV structure with IQ we used restricted cubic splines with four knots placed at the 5th, 35th, 65th, and 95th percentiles, with the 10th percentile set as the reference. Potential non-linearity was tested using a likelihood ratio test.

Covariates adjusted in the analysis included maternal age (continuous), maternal educational level (high school and below, college/bachelor and above), maternal annual income (< 100,000 CNY or ≥ 100,000 CNY), offspring gender (male/female) and body mass index (BMI) at 4-years of age (continuous). Missing values for categorical variables were considered as a separate category. To assess the robustness of our findings, sensitivity analyses were performed after excluding children with obesity or those born preterm.

All analyses were performed using Stata version 14.0 (StataCorp, College Station, TX, USA) and R version 4.2.0 (R Foundation for Statistical Computing, Vienna, Austria) using 2-sided tests with a *P* value < 0.05 considered statistically significant.

## Results

### Basic cohort characteristics

Amongst the 1279 children included in this study, 52.5% (*n* = 672) were male. Mean (SD) systolic BP (SBP), diastolic BP (DBP), RWT and LVMI at 4 years of age were 98.3 (7.7) mmHg, 57.4 (6.3) mmHg, 22.5% (3.3%) and 26.2 (4.8) g/m^2.7^, respectively (Table 1). Mean (SD) maternal age at delivery was 30.9 (3.5) years. 71.2% (*n* = 815) of mothers had a Bachelor’s degree or above and 37.4% (*n* = 478) families had an annual income over 100,000 RMB. Approximately 20.7% (*n* = 265) of recruited children had elevated BP, and 5.2% (*n* = 67) had LVH.

**Table 1:** Baseline maternal characteristics and cardiovascular parameters of their children (*N* = 1279)

Variables	Values
Maternal age (y)	30.9 ± 3.5
Pre-pregnancy BMI (kg/m^2^)	22.0 ± 3.3
Maternal education	
Junior college and below	300 (23.5)
Bachelor and above	815 (63.7)
Unknown or missed	164 (12.8)
Paternal education	
Junior college and below	281 (22.0)
Bachelor and above	783 (61.2)
Unknown	215 (16.8)
Annual family income (1000 CNY)	
< 100	315 (24.6)
≥ 100	478 (37.4)
Unknown	486 (38.0)
Child sex	
Male	672 (52.5)
Female	607 (47.5)
Gestational age (wk)	39.1 ± 1.5
Weight at 4-y-old (kg)	17.9 ± 2.8
Height at 4-y-old (cm)	108.3 ± 4.8
BMI at 4-y-old (kg/m^2^)	15.2 ± 1.7
SBP at 4-y-old (mmHg)	98.3 ± 7.7
DBP at 4-y-old (mmHg)	57.4 ± 6.3
RWT at 4-y-old (%)	22.5 ± 3.3
LVMI at 4-y-old (g/m^2.7^)	26.2 ± 4.8
Elevated BP in 4-y-old (%)	265 (20.7)
LVH in 4-year-old (%)	67 (5.2)

Values are mean ± standard deviation or *n* (%). *BMI* body mass index, *BP* blood pressure, *SBP* systolic BP, *DBP* diastolic BP, *RWT* relative wall thickness, *LVMI* left ventricular mass indexed to the height in m^2.7^, *LVH* left ventricular hypertrophy

### WPPSI-IV scores stratified by left ventricular hypertrophy and blood pressure

Mean (SD) FSIQ was 115.9 (11.6), and subscale IQ included PSI 106.6 (10.8), WMI 105.4 (12.3), VCI 117.1 (13.1), VSI 114.5 (14.0) and FRI 110.5 (12.7) (Table 2). Compared to children with normal BP and non-LVH, children in the elevated BP and non-LVH group had lower FSIQ (113.0 ± 11.7 versus 116.6 ± 11.5), WMI (103.3 ± 12.0 versus 106.0 ± 12.4), VCI (114.9 ± 14.2 versus 117.8 ± 12.7), and VSI (111.7 ± 13.2 versus 115.2 ± 14.1) (Table 2). The WPPSI-IV score in children with LVH was not significantly different from the non-LVH group (*P* > 0.05), except for VCI (117.2 ± 13.1 versus 114.6 ± 14.8, Supplementary Table 1).

**Table 2:** WPPSI-IV scores of 4-year-old children stratified by blood pressure and LVMI status

Variables	Total (*N* = 1279)	Normal BP and non-LVH (*n* = 961)	Normal BP and LVH (*n* = 53)	Elevated BP and non-LVH (*n* = 251)	Elevated BP and LVH (*n* = 14)	*P*
Full-scale IQ	115.9 ± 11.6	116.6 ± 11.5	114.8 ± 12.0	113.0 ± 11.7^*^	117.0 ± 8.23	<** 0**.**001**
Processing speed index	106.6 ± 10.8	107.1 ± 10.7	105.0 ± 10.9	105.4 ± 11.2	106.0 ± 10.2	0.104
Working memory index	105.4 ± 12.3	106.0 ± 12.4	104.2 ± 12.1	103.3 ± 12.0^*^	103.5 ± 13.4	**0**.**014**
Verbal comprehension index	117.1 ± 13.1	117.8 ± 12.7	115.2 ± 13.7	114.9 ± 14.2^*^	119.9 ± 11.8	**0**.**009**
Visual spatial index	114.5 ± 14.0	115.2 ± 14.1	113.4 ± 13.5	111.7 ± 13.2^*^	115.9 ± 11.3	**0**.**005**
Fluid reasoning index	110.5 ± 12.7	110.9 ± 12.7	110.7 ± 13.4	108.9 ± 12.5	110.7 ± 11.5	0.186

Values are means ± standard deviation. *BP* blood pressure, *LVMI* left ventricular mass indexed to the height in m^2.7^, *IQ* intelligence quotient, *LVH* left ventricular hypertrophy. The *P* column shows the *P* value from one-way analysis of variance across the four groups, bold *P* values indicate *P* < 0.05.  ^*^P < 0.05 compared with normal BP and non-LVH group in Bonferroni post hoc analysis

### Independent association of blood pressure with cognitive development in children

With children grouped by BP and LVMI we used linear regression to examine potential associations. Compared to the normal-BP and non-LVH group, the elevated BP and non-LVH group was significantly associated with lower FSIQ [adjusted mean difference (*β)* = − 3.28, 95% confidence interval (CI) = − 5.02, − 1.53] and all subscale IQ scores, including PSI (*β* = − 2.10, 95% CI = − 3.78, − 0.43), WMI (*β* = − 2.97, 95% CI = − 4.93, − 1.01), VCI (*β* = − 2.06, 95% CI = − 4.06, − 0.63), VSI (*β* = − 3.13, 95% CI = − 5.28, − 0.99), and FRI (*β* = − 2.00, 95% CI = − 3.95, − 0.05) (Table 3). There was no significant additive interaction between elevated BP and LVH (*P* > 0.05) even after further adjustment for confounders (Supplementary Table 2).

**Table 3:** Adjusted differences in WPPSI-IV scores at different blood pressures and LVMI status in 4-year-old children

Variables	Normal BP and non-LVH	Normal BP and LVH	Elevated BP and non-LVH	Elevated BP and LVH
Full-scale IQ	Reference	− 1.22 (− 4.39, 1.93)	**− 3.28 (− 5.02, − 1.53)**	1.75 (− 4.84, 8.35)
Processing speed index	Reference	− 2.14 (− 5.17, 0.89)	**− 2.10 (− 3.78, − 0.43)**	2.00 (− 4.32, 8.31)
Working memory index	Reference	− 1.39 (− 4.94, 2.15)	**− 2.97 (− 4.93, − 1.01)**	− 0.84 (− 8.23, 6.56)
Verbal comprehension index	Reference	− 1.70 (− 5.31, 1.91)	**− 2.06 (− 4.06, − 0.63)**	1.15 (− 6.39, 8.68)
Visual spatial index	Reference	− 1.62 (− 5.51, 2.26)	**− 3.13 (− 5.28, − 0.99)**	4.53 (− 3.58, 12.64)
Fluid reasoning index	Reference	− 0.21 (− 3.73, 3.32)	**− 2.00 (− 3.95, − 0.05)**	2.75 (− 4.60, 10.11)

Values are adjusted mean differences (*β*) with 95% confidence intervals from linear regression models, representing differences in WPPSI-IV scores compared with the normal BP and non-LVH group. *WPPSI-IV* Wechsler primary and preschool scales of intelligence-fourth edition, *BP* blood pressure, *LVMI* left ventricular mass indexed to the height in m^2.7^, *LVH* left ventricular hypertrophy, *IQ* intelligence quotient. Bold values indicate *P* < 0.05, corresponding to 95% confidence intervals that do not include 0. Models were adjusted for maternal age, maternal annual income, maternal education level, offspring sex, and body mass index

To further explore the independent role of BP, we conducted linear regression with normal-BP as a reference adjusting, for LVMI. Elevated BP remained significantly associated with lower FSIQ (*β* = − 2.94, 95% CI = − 4.65, − 1.24), PSI (*β* = − 1.76, 95% CI = − 3.39, − 0.12), WMI (*β* = − 2.77, 95% CI = − 4.68, − 0.86), and VSI (*β* = − 2.64, 95% CI = − 4.74, − 0.55) (Supplementary Table 3). Across LVMI groups, SBP was negatively associated with VSI (*β* = − 0.22, 95% CI = − 0.43, − 0.02) in children in the highest LVMI tertile. A similar negative association was also observed in children in the first or second LVMI tertile; this was not statistically significant (Supplementary Table 4). Mediation analysis showed no significant indirect effect of LVMI on the association between BP levels and WPPSI-IV scores, indicating that this association was not mediated by LVMI (Supplementary Table 5).

### Association between blood pressure, left ventricular structure and cognitive development

**Fig. 1:**
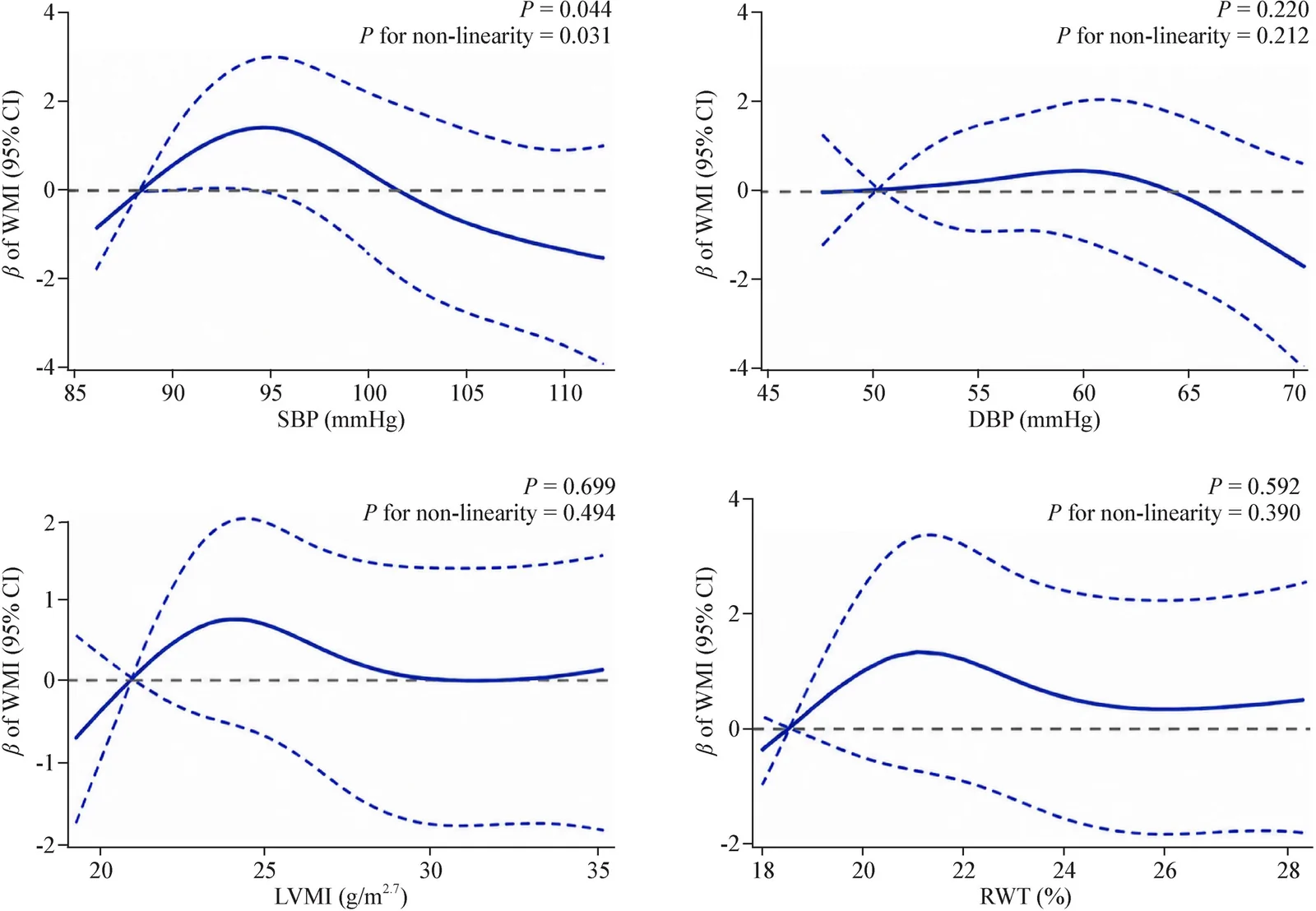
Restricted cubic spline relationship of blood pressure and cardiac structure with working memory index. Models were adjusted for maternal age, economic and educational levels, offspring sex, and body mass index. The solid blue line represents the estimated *β*, the blue dashed lines represent the 95% confidence interval, and the gray horizontal dashed line represents the reference line at *β* = 0. The *P* value indicates the overall association from the restricted cubic spline model, and the *P* for non-linearity indicates evidence of non-linearity. *WMI* working memory index, *SBP* systolic blood pressure, *DBP* diastolic blood pressure, *LVMI* left ventricular mass indexed to the height in m^2.7^, *RWT* relative wall thickness, *CI* confidence interval

**Fig. 2:**
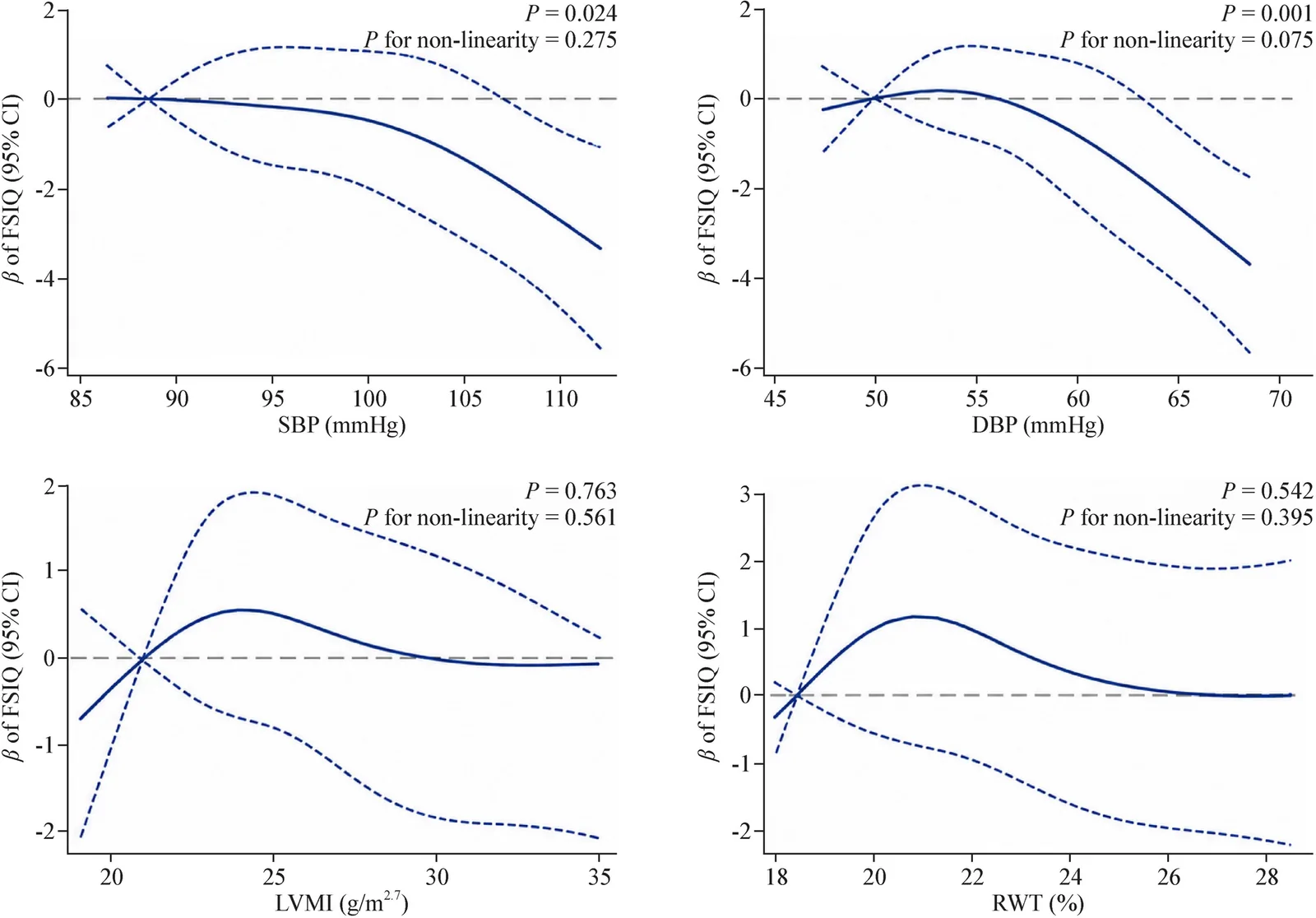
Restricted cubic spline relationship of blood pressure and cardiac structure with full-scale intelligence quotient. Models were adjusted for maternal age, economic and educational levels, offspring gender and body mass index. The solid blue line represents the estimated *β*, the blue dashed lines represent the 95% confidence interval, and the gray horizontal dashed line represents the reference line at *β* = 0. The *P* value indicates the overall association from the restricted cubic spline model, and the *P* for non-linearity indicates evidence of non-linearity. *FSIQ* full-scale intelligence quotient, *SBP* systolic blood pressure, *DBP* diastolic blood pressure, *LVMI* left ventricular mass indexed to the height in m^2.7^, *RWT* relative wall thickness, *CI* confidence interval

Multiple linear regression and restricted cubic spline modelling was used to further explore the association between BP, LV structure and cognitive development. Children with higher BP, either SBP or DBP, were more likely to have lower WPPSI-IV scores in early childhood. There was a significant negative association between both SBP and DBP and FSIQ in early childhood. With further adjustment for maternal age, economic and educational levels, offspring gender and BMI, FSIQ still decreased significantly as SBP (*β* = − 0.12, 95% CI = − 0.21, − 0.03) and DBP (*β* = − 0.16, 95% CI = − 0.26, − 0.05) increased. In subscale IQ scores, lower VSI was significantly associated with increased SBP (*β* = − 0.23, 95% CI = − 0.35, − 0.10) and DBP (*β* = − 0.23, 95% CI = − 0.35, − 0.11) (Table 4). In addition, there was an inverted U-shaped association showed between SBP and WMI (*P*_non-linearity_ = 0.031) (Fig. 1). Using a recursive algorithm, we identified 94 mmHg as the inflection point at which the direction of the association changed. There was a negative association between SBP and WMI when SBP was over 94 mmHg (*β* = − 0.20, 95% CI = − 0.36, − 0.05), whereas no significant association was observed when SBP was under 94 mmHg (*β* = 0.18, 95% CI = − 0.22, 0.59) (Supplementary Table 6). Similar results were observed using 95 mmHg as the cutoff. There was no significant association using age-, sex-, and height-specific 90th or 95th thresholds. There was no non-linear association between BP and FSIQ or other subscale WPPSI-IV scores **(***P*_non-linearity_ > 0.05; Fig. 2 and Supplementary Figs. 1–4).

**Table 4:** Association of blood pressure and cardiac structure with WPPSI-IV scores in 4-year-old children

Variables	Crude model				Adjusted model			
	SBP (mmHg)	DBP (mmHg)	RWT (%)	LVMI (g/m^2.7^)	SBP (mmHg)	DBP (mmHg)	RWT (%)	LVMI (g/m^2.7^)
Full-scale IQ	**− 0.12 (− 0.20, − 0.04)**	**− 0.15 (− 0.25, − 0.05)**	− 0.17 (− 0.36, 0.18)	− 0.09 (− 0.22, 0.04)	**− 0.12 (− 0.21, − 0.03)**	**− 0.16 (− 0.26, − 0.05)**	0.01 (− 0.18, 0.21)	− 0.04 (− 0.10, 0.17)
Processing speed index	− 0.05 (− 0.13, 0.02)	0.002 (− 0.09, 0.09)	− 0.08 (− 0.26, 0.09)	− 0.09 (− 0.21, 0.04)	− 0.06 (− 0.15, 0.03)	− 0.06 (− 0.16, 0.04)	− 0.002 (− 0.19, 0.18)	− 0.02 (− 0.15, 0.11)
Working memory index	− 0.06 (− 0.15, 0.02)	− 0.04 (− 0.14, 0.06)	− 0.04 (− 0.24, 0.16)	− 0.06 (− 0.20, 0.08)	− 0.07 (− 0.18, 0.05)	− 0.06 (− 0.17, 0.04)	0.08 (− 0.14, 0.30)	0.03 (− 0.13, 0.18)
Verbal comprehension index	− 0.08 (− 0.17, 0.01)	**− 0.12 (− 0.23, − 0.01)**	− 0.20 (− 0.42, 0.01)	− 0.14 (− 0.28, 0.01)	− 0.09 (− 0.21, 0.03)	− 0.11 (− 0.23, − 0.002)	− 0.05 (− 0.28, 0.17)	− 0.04 (− 0.20, 0.11)
Visual spatial index	**− 0.13 (− 0.23, − 0.04)**	**− 0.20 (− 0.32, − 0.09)**	**− 0.28 (− 0.51, − 0.06)**	− 0.11 (− 0.26, 0.05)	**− 0.23 (− 0.35, − 0.10)**	**− 0.23 (− 0.35, − 0.11)**	− 0.13 (− 0.38, 0.11)	− 0.01 (− 0.16, 0.18)
Fluid reasoning index	− 0.08 (− 0.16, 0.01)	− 0.09 (− 0.20, 0.01)	− 0.19 (− 0.40, 0.01)	− 0.01 (− 0.15, 0.13)	− 0.09 (− 0.20, 0.03)	− 0.11 (− 0.22, 0.01)	0.01 (− 0.23, 0.21)	0.12 (− 0.04, 0.27)

Values are regression coefficients (*β*) with 95% confidence intervals from linear regression models, representing the mean difference in WPPSI-IV scores per one-unit increase in SBP, DBP, RWT, or LVMI. *WPPSI-IV* Wechsler primary and preschool scales of intelligence-fourth edition, *SBP* systolic blood pressure, *DBP* diastolic blood pressure, *RWT* relative wall thickness, *LVMI* left ventricular mass indexed to the height in m^2.7^, *IQ* intelligence quotient. Bold values indicate *P* < 0.05, corresponding to 95% confidence intervals that do not include 0. Adjusted model: adjusted for offspring sex (male/female), body mass index, maternal age, maternal annual income (< 100,000 CNY or ≥ 100,000 CNY) and education level (high school and below, college/bachelor and above)

With respect to LV structure, FSIQ was not associated with RWT (*β* = − 0.01, 95% CI = − 0.18, 0.21) or LVMI (*β* = − 0.04, 95% CI = − 0.10, 0.17) in linear regression analysis. Other subscale scores were also not significantly associated with LV structure (Table 4). Similar results were seen in restrictive cubic spline modelling (Supplementary Figs. 1–4).

### Sensitivity analysis

To examine the robustness of our findings, sensitivity analysis was undertaken in children without obesity and children born at term. Elevated BP remained significantly associated with poorer cognitive performance and was independent of LV structure (Supplementary Tables 7 and 8). Similar associations between higher BP and lower FSIQ and VSI were still observed (Supplementary Tables 9 and 10). In addition, we repeated the analyses using the 95th percentile of SBP as an alternative threshold for elevated SBP (Supplementary Tables 11–12) and the 90th percentile of LVMI as an alternative cutoff for increased LVMI (Supplementary Tables 13–14); our findings remained materially unchanged.

## Discussion

The heart–brain connection has attracted much attention in recent years. Although increasing research shows that hypertension increases the risk of cognitive impairment, the role of LV remodeling in this association remains undetermined. We demonstrated that elevated BP was associated with lower cognitive performance in young children and that was independent of LVMI.

Hypertension in adolescents and young adults has been linked to lower cognitive function in adolescence, young adulthood [2, 29] and in mid-life [30]. This provides some further biological plausibility to our findings. Of note, although elevated BP was associated with lower cognitive scores, the differences were modest, remained within the normal range of neurocognitive tests, and did not indicate overt cognitive impairment. This is consistent with previous findings in hypertensive youths [31]. Our study extended these observations to preschool-aged children, indicating that early elevations in SBP are also associated with subtle alterations in brain function. In early life, the link between elevated BP and lower cognition may be partly related to alterations in neurovascular coupling, cerebral autoregulation, or subclinical cerebral hypoperfusion during this developmental stage [32]. Interestingly, an exploratory inverted U-shaped relationship was observed between SBP and WMI, with an estimated inflection point of 94 mmHg. Although this finding was not confirmed using P90 or P95 thresholds, it provides preliminary evidence warranting further investigation [19]. These findings extend our current understanding of heart–brain connections to an earlier stage of life.

Most previous studies have described decreased cognitive function as a central hypertensive target. However, whether more intensive BP management would benefit specific patient populations is inconclusive [33]. Encouraging results from the SPRINT-MIND study shows that an intensive BP control may prevent mild cognitive impairment in the elderly [34]. However, a newly published study demonstrated that while most individuals benefit from intensive BP control, this cannot reduce the prevalence of dementia [35]. Critically, the optimal range for BP control remains undetermined. Current pediatric guidelines derive thresholds from epidemiological percentiles rather than outcome-based evidence. Our findings suggest that SBP within an ideal physiological range may be associated with better early cognitive development, but interventional studies in children are needed to establish causality and evidence-based BP targets.

Whilst several previous studies in adults have indicated that LV remodeling is linked to an increased risk of cognitive impairment, the present study shows no such association between LV structure and cognitive development in children. The discrepancy in the young population provides clues for the exploration of possible mechanisms underlying heart–brain connections. As a time-integrated marker of exposure to hypertension and several cardiovascular risk factors in adults [36, 37], LVH is considered a potential early indicator for cognitive decline in the hypertensive population. Previous studies conducted in hypertensive older adults reported a correlation between increased LVM or LV remodeling and cognitive dysfunction and dementia [6, 38, 39]. The effect of LVMI on the association between BP and cognitive function has been quantified by Razavi et al. [11]. They found that nearly 20% of the relationship between life-long BP burden and cognitive decline was mediated by LV remodeling.

In our study the association between BP and cognitive function in young children was not mediated by LVMI or LVH. Compared to previous studies, our participants were unselected young children free from cardiovascular or other chronic diseases. The lack of association between LV structural changes and cognitive performance in 4-year-old children suggest that the correlation between LV remodeling and impaired cognitive function may partly reflect shared underlying vascular or other residual factors, rather than a direct effect of LV remodeling on cognitive impairment. Potential mechanisms linking LVMI and cognitive function may include altered hemodynamics due to abnormal LV structure [40, 41] leading to subsequent cerebrovascular dysregulation [42]. Indeed, it has been reported that LV remodeling is associated with cerebral small vessel diseases [9] and brain atrophy [43]. In addition, previous adult studies have reported that the association between LVM and cognitive decline is attenuated after adjustment for cardiovascular disease [8, 43], supporting a role for vascular pathology in mediating this response.

Several studies have documented that the association between LVMI and lower cognitive performance was independent of blood pressure or large artery stiffness [6, 8]. In a systematic review and meta-analysis, Papadopoulos et al. also found that LVH was associated with neuroimaging markers of cerebral small vessels disease independent of hypertension and other vascular risk factors [9]. This independent association could be explained by residual confounding, since there may be insufficient adjustments for duration of high BP in spite of hypertension control, especially in cross-sectional studies. Another possible explanation for the independent association could be ascribed to the fact that, both LV structure and cognition are sensitive indicators of lifelong exposure to BP [44]. A birth cohort from the United Kingdom found that, even after accounting for existing BP, elevated BP during early midlife predicted higher LVMI in later life. This was the case even in those receiving antihypertensive medication, suggesting that cardiac damage might not be completely reversible [45].

Compared to previous studies, our participants were very young. As a gradual adaption to pressure overload, LV remodeling in children usually presents as a physiological change without impaired cardiac function [46, 47]. The young age of our participants and their relatively short exposure to LV structural changes may partly explain why no association with cognitive performance was observed. Unlike adults, young children may not have experienced pressure overload of sufficient duration or severity to result in LVH of a degree capable of affecting cerebral perfusion or cognitive function. In our follow-up study of the offspring at 7 and 10 years of age, this question might be answered. The present findings of no association between LV structural changes and cognitive performance at 4 years of age highlight the importance of age-specific mechanisms in the heart–brain axis.

Elevated BP and increased LVMI often coexist in the elderly, leading to an overlapping target organ effect. Previous studies in adults struggle to define an independent association between BP and LV structure for several reasons; these include aging, vascular dysfunction and cardiovascular events. By focusing on a relatively homogeneous cohort of preschoolers in the absence of aging-related comorbidities, this study is the first to examine the distinct contributions of SBP and LV remodeling on cognitive development in early childhood. We demonstrate that higher SBP levels are associated with lower cognitive function independent of LV remodeling.

The study has some limitations. First, it is cross-sectional study and cannot establish causality. Longitudinal studies are needed to confirm temporal and dose–response relationships. Second, the SBC families are mostly from urban areas and have relatively high socioeconomic status, thereby limiting the generalizability of our findings to other areas and lower socio-economic groups. Third, although our cohort was ostensibly healthy, using current normative thresholds 20.7% met the criteria for elevated blood pressure. This suggests that existing BP percentiles may not be optimal for Chinese preschoolers, and population-specific, outcome-based thresholds should be developed. Future studies using 24-h ambulatory BP monitoring are warranted to better characterize BP burden and variability, which may be more closely related to LVMI and cerebral vascular damage than snapshot BP alone [48, 49].

In conclusion, in this cross‐sectional study of urban Chinese preschoolers, elevated SBP showed a modest association with lower performance in cognitive development; this was independent of left ventricular remodeling. By addressing this association in a population free from cardiovascular risk factors, we provide experience in young children that may facilitate multidisciplinary approaches for optimal blood pressure evaluation and management. Our findings underscore the need to maintain ideal BP during early life. Prospective studies with longitudinal follow-up in diverse pediatric populations are warranted to translate these findings into clinical BP targets to optimize early cognitive development.

## Supplementary Information

Below is the link to the electronic supplementary material.

Supplementary file 1 (DOCX 3325 KB)

## Data Availability

The datasets generated and/or analyzed during the current study are available from the corresponding author on reasonable request.
