# Supplementary material for: Blood pressure, left ventricular structure and cognitive development in young children: a cross-sectional study
Source: World J Pediatr. 2026 Aug 14;22(7):782–92. doi: 10.1007/s12519-026-01078-7 (PMC13577968; doi:10.1007/s12519-026-01078-7)
Supplement: Supplementary file 1 [file 12519_2026_1078_MOESM1_ESM.docx]

**Supplementary materials**

**Supplementary Table 1**. Comparison of WPPSI-IV scores of 4-year-old children with differing BP and LVMI values

| **Variables** | **Total (*N* = 1279)** | **NBP (*n* = 1014)** | **EBP (*n* = 265)** | ***P*** | **Non-LVH (*n* = 1212)** | **LVH (*n* = 67)** | ***P*** |
| --- | --- | --- | --- | --- | --- | --- | --- |
| Full-scale IQ | 115.9 ± 11.6 | 116.5 ± 11.5 | 113.3 ± 11.6 | **< 0.001** | 115.9 ± 11.7 | 114.0 ± 13.6 | 0.071 |
| Processing speed index | 106.6 ± 10.8 | 107.0 ± 10.7 | 105.4 ± 11.1 | **0.037** | 106.6 ± 10.9 | 105.3 ± 12.1 | 0.182 |
| Working memory index | 105.4 ± 12.3 | 105.9 ± 12.4 | 103.3 ± 12.1 | **0.002** | 105.5 ± 12.3 | 104.2 ± 13.4 | 0.247 |
| Verbal comprehension index | 117.1 ± 13.1 | 117.7 ± 12.8 | 115.1 ± 14.1 | **0.005** | 117.2 ± 13.1 | 114.6 ± 14.8 | **0.031** |
| Visual spatial index | 114.5 ± 14.0 | 115.1 ± 14.1 | 112.0 ± 13.1 | **< 0.001** | 114.6 ± 14.0 | 112.7 ± 14.7 | 0.126 |
| Fluid reasoning index | 110.5 ± 12.7 | 110.9 ± 12.7 | 109.0 ± 12.4 | **0.033** | 110.5 ± 12.5 | 110.2 ± 14.6 | 0.780 |

Values are means ± standard deviation. The *P* columns show *P* values from two-sided Student’s *t* tests comparing NBP with EBP and LVH with non-LVH, respectively. The bold font indicates *P* < 0.05. *WPPSI-IV* Wechsler primary and preschool scales of intelligence-fourth edition, *BP* blood pressure, *LVMI* left ventricular mass indexed to the height in m^2.7^, *NBP* normal BP, *EBP* elevated BP, *LVH* left ventricular hypertrophy, *IQ* intelligence quotient

**Supplementary Table 2**. Test for synergistic interaction between SBP and LVMI on WPPSI-IV score in children

| **Variables** | **NBP** | **EBP** |  | **Non-LVH** | **LVH** |  | **EBP*LVH** |
| --- | --- | --- | --- | --- | --- | --- | --- |
| Full-scale IQ | Reference | **− 3.28 (− 5.03, − 1.53)** |  | Reference | − 1.23 (− 4.39, 1.93) |  | 6.25 (− 1.14, 13.66) |
| Processing speed index | Reference | **− 2.10 (− 3.78, − 0.43)** |  | Reference | − 2.14 (− 5.17, 0.89) |  | 6.24 (− 0.85, 13.33) |
| Working memory index | Reference | **− 2.97 (− 4.93, − 1.10)** |  | Reference | − 1.39 (− 4.94, 2.15) |  | 3.53 (− 4.77, 11.83) |
| Verbal comprehension index | Reference | **− 2.05 (− 4.06, − 0.06)** |  | Reference | − 1.70 (− 5.31, 1.91) |  | 4.91 (− 3.55, 13.37) |
| Visual spatial index | Reference | **− 3.13 (− 5.28, − 0.99)** |  | Reference | − 1.62 (− 5.51, 2.26) |  | **0.29 (0.18, 18.39)** |
| Fluid reasoning index | Reference | **− 2.00 (− 3.95, − 0.05)** |  | Reference | − 0.21 (− 3.73, 3.32) |  | 4.96 (− 3.30, 13.22) |

Values are adjusted mean differences (*β*) with 95% confidence intervals from linear regression models. The interaction term EBP × LVH was used to assess the interaction between elevated BP and LVH. Bold values indicate *P* < 0.05, corresponding to 95% confidence intervals that do not include 0. Models were adjusted for maternal age, maternal education, annual family income, child sex, and body mass index at 4 years. *WPPSI-IV* Wechsler primary and preschool scales of intelligence-fourth edition, *LVMI* left ventricular mass indexed to the height in m^2.7^, *SBP* systolic blood pressure, *NBP* normal blood pressure, *EBP* elevated blood pressure, *LVH* left ventricular hypertrophy, *IQ* intelligence quotient

**Supplementary Table 3**. Role of LV structure on the association between BP and WPPSI-IV scores in children

| **Variables** | **Normal BP** | **Elevated BP** | | |
| --- | --- | --- | --- | --- |
|  |  | **Crude model** | **Model 1** | **Model 2** |
| Full-scale IQ | Reference | **− 3.20 (− 4.76, − 1.63)** | **−** **2.94 (− 4.65, − 1.25)** | **− 2.94 (****− 4.65, − 1.24)** |
| Processing speed index | Reference | **− 1.56 (− 3.02, − 0.09)** | **− 1.75 (− 3.38, − 0.12)** | **− 1.76 (****− 3.39, − 0.12)** |
| Working memory index | Reference | **− 2.61 (− 4.28, − 0.95)** | **− 2.77 (****− 4.68, − 0.86)** | **− 2.77 (****− 4.68, − 0.86)** |
| Verbal comprehension index | Reference | **− 2.51 (− 4.28, − 0.74)** | − 1.78 (− 3.73, 0.16) | − 1.79 (− 3.73, 0.16) |
| Visual spatial index | Reference | **− 3.20 (− 5.08, − 1.31)** | **− 2.64 (****− 4.74, − 0.55)** | **− 2.64 (****− 4.74, − 0.55)** |
| Fluid reasoning index | Reference | **− 1.86 (− 3.57, − 0.15)** | − 1.75 (− 3.65, 0.15) | − 1.74 (− 3.63, 0.16) |

Values are adjusted mean differences (*β*) with 95% confidence intervals from linear regression models, representing differences in WPPSI-IV scores compared with the normal BP group. Bold values indicate *P* < 0.05, corresponding to 95% confidence intervals that do not include 0. Model 1: adjusted for maternal age, economic and educational levels, offspring gender and body mass index; Model 2: further adjusted for LVMI. *LV* left ventricular, *WPPSI-IV* Wechsler primary and preschool scales of intelligence-fourth edition, *BP* blood pressure, *IQ* intelligence quotient, *LVMI* left ventricular mass indexed to the height in m^2.7^

**Supplementary Table 4**. Association between SBP and LVMI and WPPSI-IV score in different tertiles

| **Variables** | **SBP (mmHg)** | | |  | **LVMI** | | | |
| --- | --- | --- | --- | --- | --- | --- | --- | --- |
|  | **LVMI tertile 1** | **LVMI tertile 2** | **LVMI tertile 3** |  | **SBP tertile 1** | | **SBP tertile 2** | **SBP tertile 3** |
| Full-scale IQ | − 0.13 (− 0.29, 0.02) | − 0.13 (− 0.30, 0.02) | − 0.07 (− 0.24, 0.09) |  | 0.01 (− 0.22, 0.24) | − 0.02 (− 0.20, 0.25) | | 0.11 (− 0.15, 0.37) |
| Processing speed index | − 0.06 (− 0.21, 0.10) | − 0.07 (− 0.21, 0.07) | − 0.04 (− 0.21, 0.12) |  | 0.02 (− 0.19, 0.24) | − 0.02 (− 0.25, 0.20) | | − 0.07 (− 0.32, 0.17) |
| Working memory index | 0.03 (− 0.15, 0.22) | − 0.14 (− 0.31, 0.02) | − 0.06 (− 0.25, 0.13) |  | 0.01 (− 0.26, 0.25) | 0.02 (− 0.24, 0.28) | | 0.07 (− 0.22, 0.37) |
| Verbal comprehension index | − 0.13 (− 0.31, 0.05) | − 0.04 (− 0.23, 0.14) | − 0.03 (− 0.16, 0.22) |  | − 0.20 (− 0.44, 0.05) | 0.07 (− 0.20, 0.33) | | 0.06 (− 0.24, 0.37) |
| Visual spatial index | − 0.13 (− 0.32, 0.06) | − 0.10 (− 0.29, 0.09) | **− 0.22 (− 0.43, − 0.02)** |  | 0.10 (− 0.19, 0.38) | − 0.01 (− 0.30, 0.28) | | − 0.08 (− 0.39, 0.23) |
| Fluid reasoning index | 0.002 (− 0.17, 0.17) | − 0.09 (− 0.26, 0.09) | − 0.15 (− 0.33, 0.03) |  | 0.20 (− 0.07, 0.46) | 0.05 (− 0.20, 0.30) | | 0.12 (− 0.17, 0.40) |

Values are regression coefficients (*β*) with 95% confidence intervals from linear regression models, representing the mean difference in WPPSI-IV scores per 1-mmHg increase in SBP or per one-unit increase in LVMI within each tertile group. Bold values indicate *P* < 0.05, corresponding to 95% confidence intervals that do not include 0. Models were adjusted for maternal age, economic and educational levels, offspring gender and body mass index. *WPPSI-IV* Wechsler primary and preschool scales of intelligence-fourth edition, *LVMI* left ventricular mass indexed to the height in m^2.7^, *SBP* systolic blood pressure, *IQ* intelligence quotient

**Supplementary Table 5**. Analysis of LVMI and its association with blood pressure and cognitive outcomes

| **Exposure** | **Outcome** | **Total effect, *β* (95% CI)** | **Direct effect, *β* (95% CI)** | **Indirect effect, *β* (95% CI)** | **Proportion mediated, % (95% CI)** | ***P* for indirect effect** |
| --- | --- | --- | --- | --- | --- | --- |
| SBP | Full-scale IQ | **− 0.12 (− 0.21, − 0.02)** | **− 0.12 (− 0.21, − 0.02)** | − 0.001 (− 0.007, 0.005) | 0.80 (− 6.20, 8.90) | 0.746 |
|  | Processing speed index | − 0.06 (− 0.15, 0.03) | − 0.06 (− 0.15, 0.03) | 0.001 (− 0.004, 0.007) | − 1.50 (− 29.5, 21.1) | 0.695 |
|  | Working memory index | − 0.07 (− 0.18, 0.04) | − 0.06 (− 0.18, 0.04) | − 0.001 (− 0.008, 0.006) | 1.10 (− 30.6, 33.7) | 0.834 |
|  | Verbal comprehension index | − 0.05 (− 0.15, 0.06) | − 0.05 (− 0.15, 0.06) | 0.002 (− 0.005, 0.010) | − 3.30 (− 59.1, 49.2) | 0.611 |
|  | Visual spatial index | **− 0.15 (− 0.25, − 0.04)** | **− 0.15 (− 0.25, -0.04)** | 0.0001 (− 0.007, 0.007) | − 0.10 (− 6.30, 6.60) | 0.972 |
|  | Fluid reasoning index | − 0.09 (− 0.19, 0.02) | − 0.08 (− 0.18, 0.02) | − 0.004 (− 0.013, 0.002) | 4.30 (− 17.1, 33.6) | 0.262 |
| DBP | Full-scale IQ | **− 0.156 (− 0.27, − 0.05)** | **− 0.16 (− 0.27, − 0.05)** | − 0.001 (− 0.014, 0.016) | 0.60 (− 13.3, 12.7) | 0.892 |
|  | Processing speed index | − 0.06 (− 0.16, 0.03) | − 0.07 (− 0.17, 0.03) | 0.003 (− 0.010, 0.020) | − 4.80 (− 95.7, 83.9) | 0.633 |
|  | Working memory index | − 0.06 (− 0.19, 0.06) | − 0.06 (− 0.19, 0.06) | − 0.002 (− 0.020, 0.020) | 2.30 (− 113.2, 88.2) | 0.877 |
|  | Verbal comprehension index | − 0.09 (− 0.21, 0.03) | − 0.10 (− 0.22, 0.02) | 0.006 (− 0.010, 0.030) | − 6.10 (− 93.9, 69.9) | 0.486 |
|  | Visual spatial index | **− 0.23 (− 0.35, − 0.10)** | **− 0.23 (− 0.36, − 0.11)** | 0.003 (− 0.010, 0.020) | − 1.20 (− 11.8, 6.5) | 0.729 |
|  | Fluid reasoning index | − 0.09 (− 0.21, 0.04) | − 0.08 (− 0.20, 0.05) | − 0.01 (− 0.030, 0.010) | 11.80 (− 85.9, 128.5) | 0.241 |

Values are *β* coefficients with 95% confidence intervals. Bold values indicate *P* < 0.05, corresponding to 95% confidence intervals that do not include 0. Total effect represents the association between blood pressure and cognitive outcomes without adjustment for LVMI; direct effect represents the association after adjustment for LVMI; indirect effect represents the mediated effect through LVMI, estimated using the product-of-coefficients approach. Confidence intervals and *P* values for indirect effects were obtained from 5000 bootstrap resamples. Models were adjusted for maternal age, maternal education, annual family income, child sex, and body mass index at 4 years. *LVMI* left ventricular mass indexed to the height in m^2.7^, *SBP* systolic blood pressure, *DBP* diastolic blood pressure, *IQ* intelligence quotient, *CI* confidence interval

**Supplementary Table 6**. Association between SBP and working memory index across different SBP cut-off points

| **Variables** | **SBP categories** | ***β* (95% CI)** |
| --- | --- | --- |
| WMI | < 94 mmHg | 0.18 (− 0.22, 0.59) |
|  | > 94 mmHg | − **0.20 (**− **0.36,** − **0.05)** |
|  | < 95 mmHg | 0.20 (− 0.16, 0.57) |
|  | > 95 mmHg | − **0.22 (**− **0.39,** − **0.04)** |
|  | < 90th | 0.05 (− 0.10, 0.20) |
|  | > 90th | 0.06 (− 0.27, 0.40) |
|  | < 95th | − 0.004 (− 0.13, 0.13) |
|  | > 95th | 0.13 (− 0.42, 0.68) |

Values are regression coefficients (*β*) with 95% confidence intervals from linear regression models, representing the association between SBP and WMI within each SBP category. Bold values indicate *P* < 0.05, corresponding to 95% confidence intervals that do not include 0. Models were adjusted for maternal age, maternal education, annual family income, child sex, and body mass index at 4 years. *SBP* systolic blood pressure, *WMI* working memory index, *CI* confidence interval

**Supplementary Table 7**. The role of left ventricular structure in the association between BP and WPPSI-IV score in non-obese children

| **Variables** | **Normal BP** | **Elevated BP** | |
| --- | --- | --- | --- |
|  |  | **Adjusted model** | **Adjusted model + LVMI** |
| Full-scale IQ | Reference | **− 2.97 (− 4.74, − 1.19)** | **− 2.98 (− 4.75, − 1.20)** |
| Processing speed index | Reference | − 1.54 (− 3.24, 0.15) | − 1.54 (− 3.23, 0.16) |
| Working memory index | Reference | **− 2.96 (− 4.94, − 0.97)** | **− 2.96 (− 4.95, − 0.97)** |
| Verbal comprehension index | Reference | − 1.40 (− 3.42, 0.62) | − 1.39 (**−** 3.41, 0.63) |
| Visual spatial index | Reference | **− 3.43 (− 5.61, − 1.24)** | **− 3.42 (− 5.61, − 1.24)** |
| Fluid reasoning index | Reference | − 1.93 (− 3.92, 0.06) | − 1.96 (− 3.95, 0.03) |

Values are regression coefficients (*β*) with 95% confidence intervals from linear regression models, representing differences in WPPSI-IV scores compared with the normal BP group. Bold values indicate *P* < 0.05, corresponding to 95% confidence intervals that do not include 0. Adjusted model: adjusted for sex, maternal age, maternal annual income and education level. *WPPSI-IV* Wechsler primary and preschool scales of intelligence-fourth edition, *BP* blood pressure, *LVMI* left ventricular mass indexed to the height in m^2.7^, *IQ* intelligence quotient

**Supplementary Table 8**. The role of left ventricular structure in the association between BP and WPPSI-IV score in children born at term

| **Variables** | **Normal BP** | **Elevated BP** | |
| --- | --- | --- | --- |
|  |  | **Adjusted model** | **Adjusted model + LVMI** |
| Full-scale IQ | Reference | **− 3.17 (− 4.88, − 1.46)** | **− 3.17 (− 4.89, − 1.45)** |
| Processing speed index | Reference | − 1.55 (− 3.19, 0.09) | − 1.55 (− 3.19, 0.09) |
| Working memory index | Reference | **− 2.64 (− 4.57, − 0.71)** | **− 2.64 (− 4.57, − 0.71)** |
| Verbal comprehension index | Reference | **− 2.03 (− 3.99, − 0.07)** | **− 2.01 (− 3.96, − 0.05)** |
| Visual spatial index | Reference | **− 3.28 (− 5.39, − 1.17)** | **− 3.26 (− 5.38, − 1.15)** |
| Fluid reasoning index | Reference | − 1.81 (− 3.73, 0.11) | − 1.84 (− 3.76, 0.07) |

Values are regression coefficients (*β*) with 95% confidence intervals from linear regression models, representing differences in WPPSI-IV scores compared with the normal BP group. Bold values indicate *P* < 0.05, corresponding to 95% confidence intervals that do not include 0. Adjusted model: adjusted for sex, body mass index, maternal age, maternal annual income and education level. *BP* blood pressure, *WPPSI-IV* Wechsler primary and preschool scales of intelligence-fourth edition, *LVMI* left ventricular mass indexed to the height in m^2.7^, *IQ* intelligence quotient

**Supplementary Table 9**. Association between blood pressure, cardiac structure and WPPSI-IV score in 4-year-old non-obese children

| **Variables** | **SBP (mmHg)** | **DBP (mmHg)** | **RWT (%)** | **LVMI (g/m^2.7^)** |
| --- | --- | --- | --- | --- |
| Full-scale IQ | **− 0.11 (− 0.20, − 0.02)** | **− 0.15 (− 0.26, − 0.04)** | 0.01 (− 0.19, 0.22) | 0.02 (− 0.12, 0.17) |
| Processing speed index | − 0.03 (− 0.12, 0.05) | − 0.05 (− 0.15, 0.06) | 0.02 (− 0.18, 0.21) | − 0.02 (− 0.16, 0.11) |
| Working memory index | − 0.07 (− 0.17, 0.04) | − 0.06 (− 0.18, 0.06) | 0.10 (− 0.12, 0.33) | 0.01 (− 0.15, 0.17) |
| Verbal comprehension index | − 0.04 (− 0.15, 0.06) | − 0.08 (− 0.20, 0.04) | − 0.05 (− 0.28, 0.18) | − 0.03 (− 0.19, 0.13) |
| Visual spatial index | **− 0.15 (− 0.27, − 0.04)** | **− 0.23 (− 0.37, − 0.10)** | − 0.15 (− 0.40, 0.10) | − 0.03 (− 0.20, 0.15) |
| Fluid reasoning index | − 0.08 (− 0.18, 0.02) | − 0.09 (− 0.21, 0.04) | − 0.002 (− 0.22, 0.22) | 0.11 (− 0.05, 0.27) |

Values are regression coefficients (*β*) with 95% confidence intervals from linear regression models, representing the mean difference in WPPSI-IV scores per one-unit increase in SBP, DBP, RWT, or LVMI. Bold values indicate *P* < 0.05, corresponding to 95% confidence intervals that do not include 0. Models were adjusted for sex (male/female), maternal age, maternal annual income (< 100,000 RMB or ≥ 100,000 RMB) and education level (high school and below, college/bachelor and above). *WPPSI-IV* Wechsler primary and preschool scales of intelligence-fourth edition, *SBP* systolic blood pressure, *DBP* diastolic blood pressure, *RWT* relative wall thickness, *LVMI* left ventricular mass indexed to the height in m^2.7^, *IQ* intelligence quotient

**Supplementary Table 10**. Association between blood pressure, cardiac structure and WPPSI-IV score in 4-year-old children born at term

| **Variables** | **SBP (mmHg)** | **DBP (mmHg)** | **RWT (%)** | **LVMI (g/m^2.7^)** |
| --- | --- | --- | --- | --- |
| Full-scale IQ | **− 0.12 (− 0.21, − 0.03)** | **− 0.17 (− 0.28, − 0.07)** | 0.03 (− 0.17, 0.24) | − 0.005 (− 0.14, 0.13) |
| Processing speed index | − 0.04 (− 0.12, 0.05) | − 0.06 (− 0.16, 0.04) | 0.01 (− 0.18, 0.20) | − 0.02 (− 0.15, 0.12) |
| Working memory index | − 0.04 (− 0.14, 0.06) | − 0.05 (− 0.17, 0.07) | 0.09 (− 0.14, 0.32) | 0.003 (− 0.15, 0.16) |
| Verbal comprehension index | − 0.06 (− 0.17, 0.04) | − 0.12 (− 0.24, 0.01) | − 0.03 (− 0.26, 0.20) | − 0.07 (− 0.23, 0.08) |
| Visual spatial index | **− 0.17 (− 0.27, − 0.06)** | **− 0.27 (− 0.41, − 0.14)** | − 0.07 (− 0.32, 0.18) | − 0.04 (− 0.21, 0.13) |
| Fluid reasoning index | − 0.06 (− 0.16, 0.04) | − 0.11 (− 0.23, 0.01) | 0.01 (− 0.21, 0.24) | 0.11 (− 0.05, 0.26) |

Values are regression coefficients (*β*) with 95% confidence intervals from linear regression models, representing the mean difference in WPPSI-IV scores per one-unit increase in SBP, DBP, RWT, or LVMI. Bold values indicate *P* < 0.05, corresponding to 95% confidence intervals that do not include 0. Models were adjusted for sex (male/female), maternal age, maternal annual income (< 100,000 RMB or ≥ 100,000 RMB) and education level (high school and below, college/bachelor and above). *WPPSI-IV* Wechsler primary and preschool scales of intelligence-fourth edition, *SBP* systolic blood pressure, *DBP* diastolic blood pressure, *RWT* relative wall thickness, *LVMI* left ventricular mass indexed to the height in m^2.7^, *IQ* intelligence quotient

**Supplementary Table 11**. WPPSI-IV scores of 4-year-old children stratified by BP and LVMI status (defined by the ≥ 95th percentile cutoffs)

| **Variables** | **Total**  **(*N* = 1279)** | **Normal BP and non-LVH**  **(*n* = 1066)** | **Normal BP and LVH**  **(*n* = 56)** | **Elevated BP and non-LVH**  **(*n* = 149)** | **Elevated BP and LVH**  **(*n* = 8)** | ***P*** |
| --- | --- | --- | --- | --- | --- | --- |
| Full-scale IQ | 115.8 ± 11.6 | 116.3 ± 11.5 | 115.0 ± 11.4 | **112.0 ± 12.4**^†^ | 114.8 ± 9.21 | **0.010** |
| Processing speed index | 106.7 ± 10.8 | 107.0 ± 10.8 | 105.3 ± 10.7 | 105.2 ± 11.0 | 101.9 ± 9.13 | 0.110 |
| Working memory index | 105.4 ± 12.3 | 105.8 ± 12.3 | 103.7 ± 12.3 | **103.2 ± 12.2****^*^** | 103.0 ± 11.4 | 0.077 |
| Verbal comprehension index | 117.1 ± 13.1 | 117.5 ± 12.8 | 115.8 ± 13.3 | **114.8 ± 14.5^*^** | 119.5 ± 15.2 | 0.087 |
| Visual spatial index | 114.5 ± 14.0 | 115.0 ± 13.9 | 114.4 ± 13.4 | **110.8 ± 14.1**^†^ | 111.3 ± 10.9 | **0.007** |
| Fluid reasoning index | 110.5 ± 12.7 | 110.8 ± 12.5 | 110.6 ± 13.3 | 108.7 ± 13.4 | 110.3 ± 10.6 | 0.330 |

Values are means ± standard deviation. The *P* column shows the *P* value from one-way analysis of variance across the four groups; bold *P* values indicate *P* < 0.05. **P* < 0.100 and †*P* < 0.05 compared with the normal BP and non-LVH group in Bonferroni post hoc analysis. Hypertension is defined as blood pressure (BP) ≥ the 95th percentile. Left ventricular hypertrophy (LVH) is defined as left ventricular mass index (LVMI) ≥ the 95th percentile. *IQ* intelligence quotient

**Supplementary Table 12**. Adjusted differences in WPPSI-IV scores for varying BP and LVMI values in 4-year-old children (defined by the ≥ 95th percentile cutoffs)

| **Variables** | **Normal BP and non-LVH** | **Normal BP and LVH** | **Hypertension and non-LVH** | **Hypertension and LVH** |
| --- | --- | --- | --- | --- |
| Full-scale IQ | Reference | − 0.34 (− 3.41, 2.73) | **− 3.54 (− 5.66, − 1.42)** | − 3.19 (− 12.90, 6.51) |
| Processing speed index | Reference | − 1.40 (− 4.34, 1.54) | **− 2.23 (− 4.26, − 0.20)** | − 3.15 (− 12.45, 6.15) |
| Working memory index | Reference | − 1.44 (− 4.89, 2.00) | **− 3.12 (− 5.49, − 0.74)** | − 1.39 (− 12.28, 9.49) |
| Verbal comprehension index | Reference | − 0.53 (− 4.04, 2.98) | − 2.02 (− 4.44, 0.40) | − 3.81 (− 14.90, 7.28) |
| Visual spatial index | Reference | − 0.05 (− 3.83, 3.73) | **− 3.61 (− 6.22, − 1.01)** | − 0.68 (− 12.61, 11.25) |
| Fluid reasoning index | Reference | 0.14 (− 3.29, 3.56) | **− 2.68 (− 5.04, − 0.32)** | 3.06 (− 7.75, 13.87) |

Values are adjusted mean differences (*β*) with 95% confidence intervals from linear regression models, representing differences in WPPSI-IV scores compared with the normal BP and non-LVH group. Bold values indicate *P* < 0.05, corresponding to 95% confidence intervals that do not include 0. Hypertension is defined as blood pressure (BP) ≥ the 95th percentile. Left ventricular hypertrophy (LVH) is defined as left ventricular mass index (LVMI) ≥ the 95th percentile. Models were adjusted for maternal age, maternal annual income, maternal education level, offspring sex, and body mass index. *WPPSI-IV* Wechsler primary and preschool scales of intelligence-fourth edition, *IQ* intelligence quotient

**Supplementary Table 13**. WPPSI-IV scores of 4-year-old children in groups stratified by BP and LVMI status (defined by the ≥ 90th percentile cutoffs)

| **Variables** | **Total**  **(*N* = 1279)** | **Normal BP and non-LVH**  **(*n* = 917)** | **Normal BP and LVH**  **(*n* = 97)** | **Elevated BP and non-LVH**  **(*n* = 234)** | **Elevated BP and LVH**  **(*n* = 31)** | ***P*** |
| --- | --- | --- | --- | --- | --- | --- |
| Full-scale IQ | 115.8 ± 11.6 | 116.7 ± 11.2 | 114.3 ± 14.1 | 113.2 ± 11.8^*^ | 113.9 ± 10.2 | **< 0.001** |
| Processing speed index | 106.7 ± 10.8 | 107.1 ± 10.6 | 105.6 ± 11.9 | 105.5 ± 11.3 | 104.6 ± 9.96 | 0.094 |
| Working memory index | 105.4 ± 12.3 | 106.0 ± 12.2 | 104.8 ± 13.5 | 103.4 ± 12.1^*^ | 102.9 ± 12.3 | **0.016** |
| Verbal comprehension index | 117.1 ± 13.1 | 118.1 ± 12.4 | 113.9 ± 15.2^*^ | 114.9 ± 14.2^*^ | 117.0 ± 13.2 | **< 0.001** |
| Visual spatial index | 114.5 ± 14.0 | 115.4 ± 14.0 | 112.9 ± 15.2 | 111.9 ± 13.2^*^ | 112.1 ± 12.6 | **0.003** |
| Fluid reasoning index | 110.5 ± 12.7 | 110.8 ± 12.5 | 111.4 ± 14.5 | 109.2 ± 12.6 | 108.1 ± 11.6 | 0.181 |

Values are means ± standard deviation. Elevated blood pressure (BP) is defined as BP ≥ the 90th percentile. Left ventricular hypertrophy (LVH) is defined as left ventricular mass index (LVMI) ≥ the 90th percentile. *WPPSI-IV* Wechsler primary and preschool scales of intelligence-fourth edition, *IQ* intelligence quotient. The *P* column shows the *P* value from one-way analysis of variance across the four groups. ^*^*P* < 0.05 compared with normal BP and non-LVH group in Bonferroni post hoc analysis

**Supplementary Table 14**. Adjusted differences in WPPSI-IV scores across BP and LVMI groups in 4-year-old children (defined by the ≥ 90th percentile cutoffs)

| **Variables** | **Normal BP and non-LVH** | **Normal BP and LVH** | **Elevated BP and non-LVH** | **Elevated BP and LVH** |
| --- | --- | --- | --- | --- |
| Full-scale IQ | Reference | − 1.36 (− 3.82, 1.10) | **− 3.35 (− 5.14, − 1.56)** | − 0.94 (− 5.48, 3.61) |
| Processing speed index | Reference | − 1.06 (− 3.44, 1.31) | **− 2.09 (− 3.81, − 0.37)** | − 0.02 (− 4.37, 4.34) |
| Working memory index | Reference | − 0.67 (− 3.43, 2.10) | **− 2.96 (− 4.97, − 0.95)** | − 1.87 (− 6.97, 3.23) |
| Verbal comprehension index | Reference | **− 2.90 (− 5.71, − 0.09)** | **− 2.35 (− 4.40, − 0.31)** | − 0.04 (− 5.23, 5.15) |
| Visual spatial index | Reference | − 1.81 (− 4.84, 1.21) | **− 3.06 (− 5.27, − 0.86)** | − 1.02 (− 6.61, 4.58) |
| Fluid reasoning index | Reference | 1.01 (− 1.74, 3.75) | − 1.77 (− 3.77, 0.23) | − 0.47 (− 5.54, 4.61) |

Values are adjusted mean differences (*β*) with 95% confidence intervals from linear regression models, representing differences in WPPSI-IV scores compared with the normal BP and non-LVH group. Bold values indicate *P* < 0.05, corresponding to 95% confidence intervals that do not include 0. Elevated blood pressure (BP) is defined as BP ≥ the 90th percentile. Left ventricular hypertrophy (LVH) is defined as left ventricular mass index (LVMI) ≥ the 90th percentile. Models were adjusted for maternal age, maternal annual income, maternal education level, offspring sex, and body mass index. *WPPSI-IV* Wechsler primary and preschool scales of intelligence-fourth edition, *IQ* intelligence quotient

**Supplementary Fig. 1** Restricted cubic spline of blood pressure and cardiac structure with processing speed index. The solid blue line represents the estimated *β*, the blue dashed lines represent the 95% confidence interval (CI), and the gray horizontal dashed line represents the reference line at *β* = 0. The *P* value indicates the overall association from the restricted cubic spline model, and the *P* for non-linearity indicates evidence of non-linearity. Models were adjusted for maternal age, economic and educational levels, offspring sex, and body mass index. *SBP* systolic blood pressure, *DBP* diastolic blood pressure, *RWT* relative wall thickness, *LVMI* left ventricular mass indexed to the height in m^2.7^, *PSI* processing speed index

**Supplementary Fig. 2** Restricted cubic spline of blood pressure and cardiac structure with verbal comprehension index. The solid blue line represents the estimated *β*, the blue dashed lines represent the 95% confidence interval (CI), and the gray horizontal dashed line represents the reference line at *β* = 0. The *P* value indicates the overall association from the restricted cubic spline model, and the *P* for non-linearity indicates evidence of non-linearity. Models were adjusted for maternal age, economic and educational levels, offspring sex, and body mass index. *SBP* systolic blood pressure, *DBP* diastolic blood pressure, *RWT* relative wall thickness, *LVMI* left ventricular mass indexed to the height in m^2.7^

**Supplementary Fig. 3** Restricted cubic spline of blood pressure and cardiac structure with visual spatial index. The solid blue line represents the estimated *β*, the blue dashed lines represent the 95% confidence interval (CI), and the gray horizontal dashed line represents the reference line at *β* = 0. The *P* value indicates the overall association from the restricted cubic spline model, and the *P* for non-linearity indicates evidence of non-linearity. Models were adjusted for maternal age, economic and educational levels, offspring sex, and body mass index. *SBP* systolic blood pressure, *DBP* diastolic blood pressure, *RWT* relative wall thickness, *LVMI* left ventricular mass indexed to the height in m^2.7^, *VSI* visual spatial index

**Supplementary Fig. 4** Restricted cubic spline of blood pressure and cardiac structure with fluid reasoning index. The solid blue line represents the estimated *β,* the blue dashed lines represent the 95% confidence interval (CI), and the gray horizontal dashed line represents the reference line at *β* = 0. The *P* value indicates the overall association from the restricted cubic spline model, and the *P* for non-linearity indicates evidence of non-linearity. Models were adjusted for maternal age, economic and educational levels, offspring sex, and body mass index. *SBP* systolic blood pressure, *DBP* diastolic blood pressure, *RWT* relative wall thickness, *LVMI* left ventricular mass indexed to the height in m^2.7^, *FRI* fluid reasoning index
